# Surgical Outcomes and Learning Curve of Robotic Single-Port Vaginal Natural Orifice Transluminal Endoscopic Surgery (vNOTES) Hysterectomy Using the Da Vinci Single-Port System

**DOI:** 10.7759/cureus.102729

**Published:** 2026-01-31

**Authors:** Qiannan Yang, Daniel Y Lovell, Xiaoming Guan

**Affiliations:** 1 Obstetrics and Gynecology, Baylor College of Medicine, Houston, USA

**Keywords:** da vinci sp platform, hysterectomy, learning curve, robot-assisted, vnotes

## Abstract

Objective: This study aims to assess the surgical outcomes and learning curve of robotic single-port vaginal natural orifice transluminal endoscopic surgery (RSP-vNOTES) for hysterectomy in benign gynecologic conditions.

Materials and methods: A retrospective observational analysis was conducted of 97 consecutively enrolled patients who underwent RSP-vNOTES hysterectomy using the da Vinci (single-port) SP system between December 2023 and July 2025 at a single tertiary care center.

Results: Median procedural durations included a total operative time of 151 minutes, a port placement time of five minutes, a docking time of two minutes, a robotic console time of 78 minutes, and a hysterectomy time of 35 minutes. One case (1.0%) required conversion to a transumbilical single-incision robotic approach for management of diaphragmatic endometriosis after completion of the vaginal hysterectomy, rather than due to failure of the hysterectomy itself. Fourteen patients (14.4%) experienced perioperative complications. Cumulative sum analysis revealed that hysterectomy time stabilized after approximately 30 cases, while port placement and docking times plateaued after 43 and 37 cases, respectively.

Conclusions: These findings suggest that proficiency in RSP-vNOTES can be achieved with sequential case experience. RSP-vNOTES appears to be a safe and effective surgical approach for hysterectomy, including complex scenarios such as endometriosis. Proficiency is typically achieved after 30 cases, particularly among surgeons with prior experience in robotic-assisted single-site and vNOTES techniques.

## Introduction

In obstetrics and gynecology, hysterectomy is among the most frequently performed surgical procedures, second only to cesarean section. In the United States, it remains highly prevalent, with an estimated 600,000 procedures performed annually and an age-adjusted prevalence of 14.6% among women aged 18 years and older based on recent national survey data [1-2]. It is indicated for a variety of benign and malignant conditions, including uterine fibroids, abnormal uterine bleeding, endometriosis, pelvic organ prolapse, and gynecologic cancers [3]. Currently, five major approaches are used for hysterectomy in the management of benign gynecologic disease: abdominal hysterectomy, vaginal hysterectomy, laparoscopic hysterectomy, robot-assisted hysterectomy, and vaginal natural orifice transluminal endoscopic surgery (vNOTES) hysterectomy, with the choice of approach determined by patient factors, disease characteristics, and surgeon expertise. As minimally invasive approaches have become increasingly adopted over the past several decades, renewed interest in vaginal hysterectomy has emerged, with vNOTES playing an important role in this evolution. Vaginal hysterectomy is considered the least invasive hysterectomy method, offering superior outcomes and fewer complications compared to other approaches [4]. Accordingly, the American College of Obstetricians and Gynecologists continues to endorse the vaginal route as the preferred method when clinically appropriate [5].

An incisionless surgery highlights the aforementioned benefits through the vagina. In conventional vaginal surgery, there can be several limitations, largely encumbered by poor visualization. With bounds in robotic technologies, hurdles encountered with conventional vaginal surgery are overcome. The integration of robot-assisted surgery with vNOTES (RA-vNOTES) has become increasingly popular for a wide range of gynecologic pathologies [6-7]. This has been recognized not only by surgeons but also by industry, with the advent of robotic platforms explicitly designed for single-incision surgery. The da Vinci single-port (SP) system (Intuitive Surgical Inc., Sunnyvale, CA, USA) was first used in 2018, initiating a new trend in robotic design. An attempt to emulate this design is also taking place in China, with two single-port robotic platforms in use: the EDGE SP1000 (Jingfeng, Shenzhen, China) and the Shurui models. The use of these platforms has been reported primarily as case reports, video articles, and case series on procedures such as sacrocolpopexy, ovarian cystectomy, and hysterectomy [8-13]. A recent study comparing RA-vNOTES hysterectomy performed using either the robotic multi-port (RMP) or robotic single-port (RSP) platform demonstrated that both approaches are safe and effective. However, the RSP platform offered logistical and ergonomic advantages and facilitated the performance of more complex procedures, particularly those requiring endometriotic excision [14]. These publications highlight the surgical precision and enhancement provided, and needed, for vNOTES. However, studies on RSP-vNOTES remain limited.

The ability to perform these procedures did not come overnight and requires prerequisite understanding in vaginal surgery and single-incision surgery for improved adaptability. Understanding the learning curve and optimizing clinical outcomes for these emerging techniques remain critical research priorities to ensure their safe and effective integration into clinical practice. Notably, the learning curve for performing hysterectomy via RSP-vNOTES has not been characterized in the current literature. Therefore, this observational, exploratory study aims to assess the learning curve and surgical outcomes of RSP-vNOTES hysterectomy for benign gynecologic indications.

## Materials and methods

This observational and retrospective study analyzed the medical records of 97 consecutively enrolled patients who underwent RSP-vNOTES hysterectomy for benign gynecologic indications between November 2023 and July 2025 at a Baylor College of Medicine-affiliated hospital. Inclusion criteria were all cases in which a hysterectomy was performed according to the standardized surgical procedure [8,15]. Cases were excluded if complete obliteration of the cul-de-sac precluded hysterectomy using the standardized surgical technique. Electronic medical records were accessed via a secure, password-protected system to extract clinical information. Patient characteristics comprised age at surgery, body mass index (BMI), ethnicity, tobacco use, history of vaginal delivery, and prior pelvic surgeries. Primary outcomes were hysterectomy time, robot docking time, port placement time, and total operative time. Secondary outcomes comprised concomitant procedures performed alongside hysterectomy, estimated blood loss, same-day discharge rate, conversion rate, uterine weight, and perioperative pain scores. Surgical complications were categorized according to the Clavien-Dindo classification system. Perioperative pain was assessed using a 10-point visual analog scale. Pain scores were obtained from preoperative clinic documentation and from a standardized patient-reported questionnaire administered during postoperative follow-up, with assessments at postoperative weeks one to three and at six weeks. Complications were recorded during the intraoperative period and within the six-week postoperative window. The need for consent to participate was waived for the retrospective nature of the study by the Institutional Review Board (IRB). This study was IRB-approved by Baylor College of Medicine on March 8, 2022, under the approval number H-51429.

The RSP-vNOTES hysterectomy protocol was standardized across all cases. X. Guan, a fellowship-trained surgeon who specializes in minimally invasive gynecologic treatments, performed all operations. This ensured that the surgical technique was consistent. Prior to the commencement of the vaginal hysterectomy, cystoscopy was performed to facilitate the placement of temporary bilateral ureteral stents, followed by the administration of indocyanine green [16]. After removal of the cystoscope, conventional vaginal hysterectomy steps were taken up to the ligation of the bilateral uterine artery pedicles. At that point, the SP access port kit (Intuitive Surgical), designed to accommodate three instruments and the robotic camera, was placed using the “4-P” port anchoring method, and the da Vinci SP robotic system was then docked [17]. The remainder of the hysterectomy, along with any additional indicated procedures, was completed with robotic assistance. Concomitant procedures, including endometriosis excision and adnexal surgery, were performed selectively based on preoperative evaluation and intraoperative findings, rather than routinely alongside hysterectomy.

Data analysis was conducted using SPSS Statistics version 25.0 (IBM Corp. Released 2017. IBM SPSS Statistics for Windows, Version 25.0. Armonk, NY: IBM Corp.) and GraphPad Prism (GraphPad Software, Inc., San Diego, CA, USA). Descriptive statistics were used to summarize the data. The Kolmogorov-Smirnov test was used first to test for continuous variables. Given the non-normal distribution of the primary outcomes, continuous variables were reported as medians with interquartile ranges (IQR), and group comparisons were conducted using the Mann-Whitney U test. Categorical data were expressed as frequencies and percentages. The learning curve for RSP-vNOTES hysterectomy was evaluated using operative time-based metrics derived from cumulative sum (CUSUM) analysis and regression modeling. CUSUM was calculated as the CUSUM of deviations of individual operative times from the overall mean, with the apex of the CUSUM plot indicating the case number at which procedural proficiency was attained. Clinical outcomes, including perioperative complications and conversion, were concurrently monitored to ensure procedural safety but were not used to define the proficiency threshold. Statistical significance was set at p < 0.05.

## Results

A total of 97 patients underwent RSP-vNOTES hysterectomy from November 2023 to July 2025. Table 1 presents the baseline patient characteristics. The median age is 39 years (IQR 34-44 years), and the median BMI is 27 kg/m² (IQR 23-31 kg/m²). Thirty-five (36.1%) patients had at least one vaginal delivery, and 67 (69.1%) patients had at least one pelvic surgery.

**Table 1:** Patient characteristics (N = 97) IQR: interquartile range, BMI: body mass index

Variables	Median (IQR) or N (%)
Age, years	39 (34-44)
BMI, kg/m^2^	27 (23-31)
Race/ethnicity	
Caucasian	62 (63.9)
African American	18 (18.6)
Hispanic	9 (9.3)
Asian	8 (8.2)
Tobacco use	9 (9.3)
Vaginal delivery history	35 (36.1)
Pelvic surgery history	67 (69.1)
Cervical surgery history	8 (8.2)
Indication for surgery	
Endometriosis	55 (56.7)
Uterine fibroids	12 (12.4)
Chronic pelvic pain	12 (12.4)
Abnormal uterine bleeding	11 (11.3)
Adenomyosis	5 (5.2)
Pelvic organ prolapse	1 (1.0)
Endometrial complex hyperplasia	1 (1.0)

**Figure 1:**
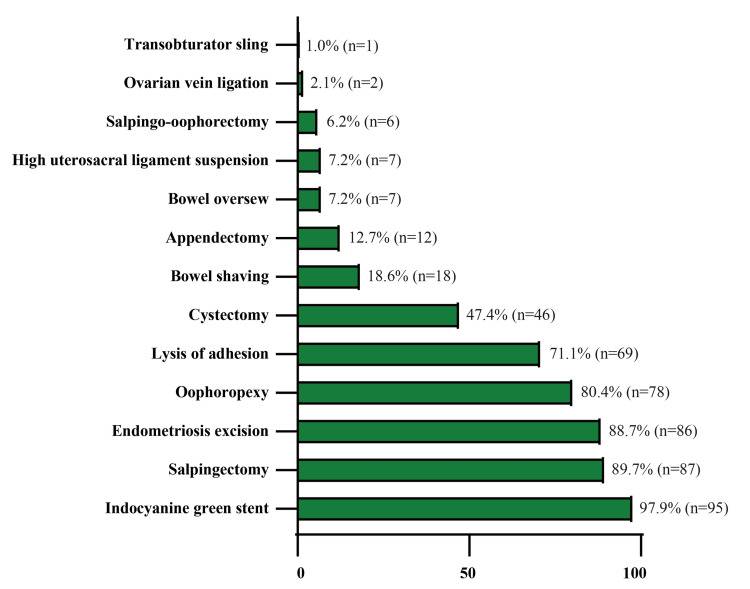
Additional surgical procedures alongside the hysterectomy Values are given as N (%).

The additional procedures performed in conjunction with the hysterectomy are shown in Figure 1. Based on pathologic findings, 86 (88.7%) patients were diagnosed with endometriosis. Based on the classification system established by the American Society for Reproductive Medicine for endometriosis, 28 (28.9%) patients had stage I, 27 (27.8%) had stage II, 23 (23.7%) had stage III, and 8 (8.2%) had stage IV disease.

Table 2 summarizes the surgical outcomes observed in the patient cohort. The median hysterectomy time was 35 minutes; robot docking time, two minutes; port placement time, five minutes; robot console time, 78 minutes; and total operative time, 151 minutes. The median uterine weight was 93 g, while the median estimated blood loss was 25 mL. The same-day discharge rate was 93.8%. One patient (1.0%) required conversion to a transumbilical robot-assisted single-incision laparoscopic approach for intraoperative management of diaphragmatic endometriosis, rather than due to failure of the hysterectomy itself.

**Table 2:** Perioperative and short-term outcomes (N = 97) IQR: interquartile range

Variables	Median (IQR) or N (%)
Hysterectomy time, min	35 (25-46)
Robot dock time, min	2 (2-3)
Port placement time, min	5 (4-6)
Robot console time, min	78 (64-107)
Total operative time, min	151 (134-184)
Estimated blood loss, mL	25 (25-50)
Same-day discharge	91 (93.8)
Conversion	1 (1.0)
Uterine weight, g	93 (65-153)

The pain scores recorded from patients before and after surgery are shown in Table 3. Compared with preoperative baseline values, lower pain scores were observed at postoperative weeks 1, 2, 3, and 6 (all p < 0.05).

**Table 3:** Perioperative pain scales (N = 97) Values are presented as median (IQR). Differences between groups were assessed using the Mann–Whitney U test, with results reported as the median difference (Hodges–Lehmann estimate with 95% CI) and the effect size (rank-biserial correlation, r). IQR: interquartile range, CI: confidence interval

Variables	Median (IQR)	Difference (95% CI)	r-value	p-value
Preoperative pain scores	8 (7-10)			
Pain score, week 1	6 (4-9)	-1 (-2, 0)	0.28	0.001
Pain score, week 2	5 (2-7)	-3 (-4, -2)	0.49	0.001
Pain score, week 3	3 (1-5)	-5 (-6, -4)	0.68	0.001
Pain score, week 6	0 (0-2)	-7 (-8, -6)	0.78	0.001

Table 4 details the overall complications, which included 14 (14.4%) cases, with 1 (1.0%) case being an intraoperative complication and 13 (13.4%) cases being postoperative complications (Clavien-Dindo grade I, II, or III complications). No instances of grade IV or V complications were identified.

**Table 4:** Surgical complications (N = 97)

Variables	N (%)
Intraoperative complications	1 (1.0)
Bladder injury	1 (1.0)
Postoperative complications	13 (13.4)
CD grade I + II	8 (8.2)
Urinary tract infection	6 (6.2)
Vaginal cuff infection	1 (1.0)
Pelvic phlegmon	1 (1.0)
CD grade III (reoperation)	5 (5.2)
Vaginal cuff reoperation	2 (2.1)
Pelvic fluid collection	2 (2.1)
Abdominal hematoma	1 (1.0)
Total	14 (14.4)

**Figure 2:**
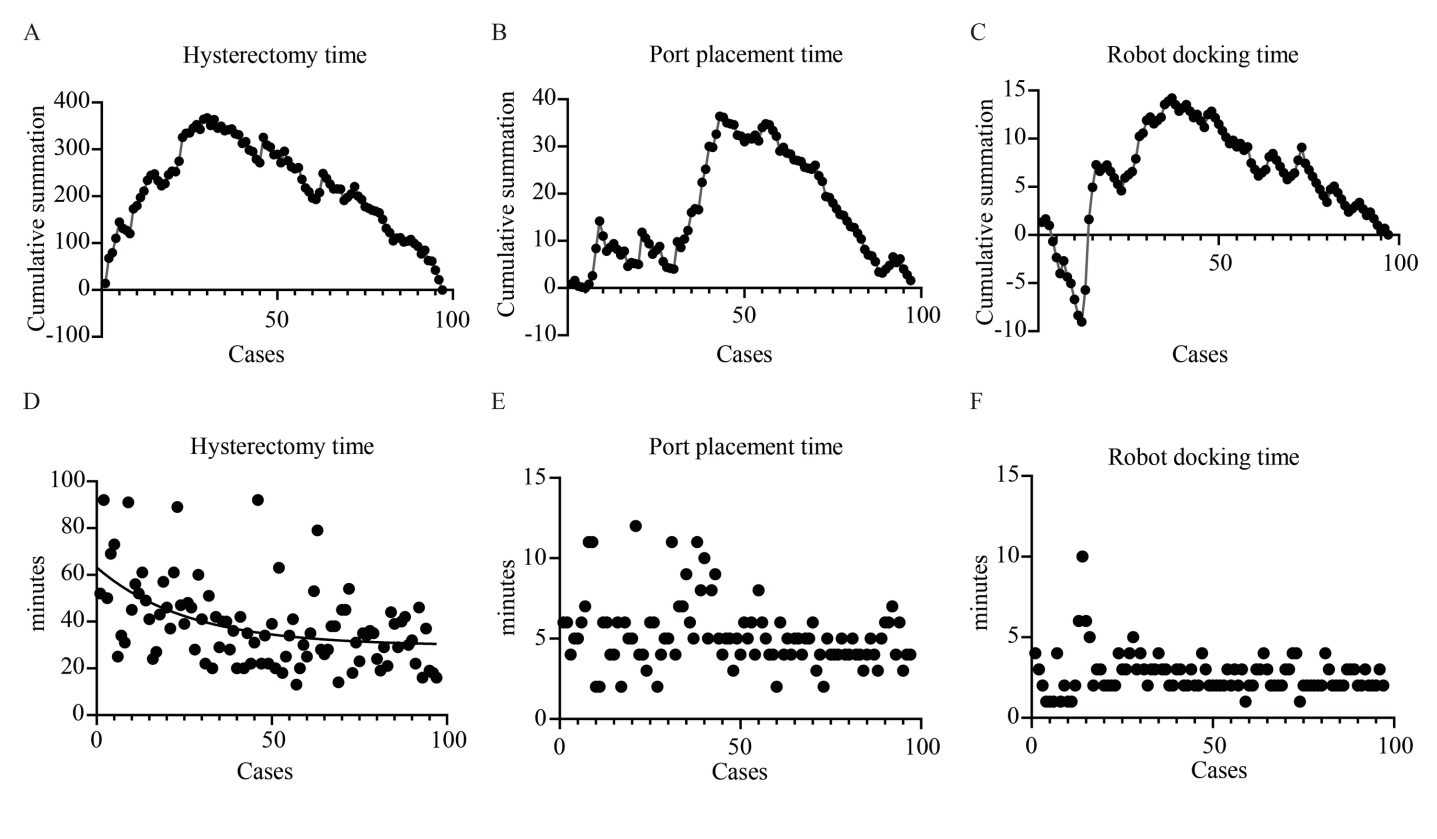
Learning curves for procedural times. (A–C) CUSUM analyses. (D–F) Scatter plots of procedural times in chronological order. Regression analysis was performed for hysterectomy time only CUSUM: cumulative sum

Subsequently, learning curves for hysterectomy time, port placement time, and robot docking time were evaluated. According to CUSUM analyses, a marked improvement in hysterectomy time was observed after approximately 30 cases, whereas port placement and robot docking times improved after approximately 43 and 37 cases, respectively, indicating the attainment of procedural proficiency at these case thresholds (Figure 2A-2C). This trend is further illustrated in Figure 2D, where the regression curve for hysterectomy time plateaus around the 30th case. However, regression analysis was not suitable for modeling port placement time and robot docking time due to the absence of a clear temporal trend and the clustering of values within a narrow range (IQR: four to six minutes for port placement time and two to three minutes for robot docking time), as shown in Figure 2E-2F.

## Discussion

Before a dedicated robotic platform for single-incision robotic surgery was developed, alternative multi-port robotic configurations were used to enable single-incision surgery. Transumbilically, the multi-port da Vinci Xi system was configured with flexible robotic instruments that passed through a dedicated port to enable single-incision surgery. This exact configuration was not applied to RA-vNOTES, but two straight robotic instruments with a robotic camera were used via two accessory ports [18-19]. This configuration introduced technical challenges, particularly collisions between the bulky robotic arms within the limited operative space between the patient’s legs. This not only hindered the utility and potential benefits of this approach but also the generalizability to other surgeons.

The introduction of the da Vinci SP system has enabled true single-site surgery, as it is specifically designed to accommodate three elbow- and wrist-mounted instruments and a flexible, angulating, high-resolution 3D camera. In the United States, the FDA has approved the da Vinci SP system for use in urology and transoral otolaryngology surgeries. The exploratory application of RSP-vNOTES in benign gynecologic surgery has been documented through a limited number of case reports [8-14]. These initial reports were optimistic about the RSP-vNOTES technology. To our knowledge, this study represents the largest case series to date and the first to evaluate the learning curve of RSP-vNOTES hysterectomy using the da Vinci SP system. Beyond prior case reports and smaller series, it advances the field by providing a standardized description of the RSP-vNOTES hysterectomy technique and a systematic characterization of its learning curve using established analytical methods. The relatively large cohort enables a more robust assessment of procedural feasibility, operative efficiency, and proficiency thresholds, thereby offering practical insights for surgeons adopting this approach.

Learning curves for surgical procedures have historically been analyzed to optimize surgical efficiency, reduce anesthesia time, improve patient outcomes, and lower healthcare costs [20-21]. To date, no published investigations have assessed the learning curve of RSP-vNOTES hysterectomy. Existing evidence suggests that proficiency in RA-vNOTES hysterectomy utilizing the da Vinci Xi system for benign gynecologic conditions is generally achieved after approximately 10 cases. In comparison, 9-50 cases were required to achieve procedural proficiency in hysterectomy via a robot-assisted transabdominal approach [6,22-26]. To minimize the influence of concomitant procedures on learning-curve assessment, the analysis was based on hysterectomy-specific operative time rather than total operative time. This approach was chosen to more effectively isolate technical learning specific to hysterectomy. However, hysterectomy time may still be influenced by variability in disease severity, including the extent of endometriosis, adhesions, and pelvic anatomy. Using this approach, the learning curves for hysterectomy time, as demonstrated by both regression and CUSUM analyses, consistently indicate that procedural proficiency in RSP-vNOTES hysterectomy was achieved after approximately 30 cases. Additionally, CUSUM analyses of port placement and robot docking times indicated that port placement time stabilized after approximately 43 cases, whereas robot docking time plateaued after 37 cases.

The learning curves for RSP and RMP vNOTES hysterectomy differ substantially due to variations in instrument handling and platform design. Surgeons with prior experience in conventional multi-port laparoscopic surgery tend to adapt more readily to the RMP-vNOTES approach, as it closely mirrors the traditional setup in terms of instrument triangulation, port distribution, and ergonomic configuration. This likely explains procedural proficiency in RMP-vNOTES within 10 cases, as the surgeon has to mainly concentrate on a different surgical vantage point through the vagina [6]. The 30 cases required to reach proficiency with the RSP-vNOTES likely reflect a steeper learning curve associated with the vaginal vantage point and the technical differences inherent in the single-port system. First, the articulated, wristed instruments utilized in RSP differ markedly from the rigid, straight instruments of RMP systems, necessitating surgical adaptation to altered tactile perception and force dynamics. Second, the SP platform's flexible, multi-jointed camera introduces additional challenges in maintaining stable visualization and achieving proper orientation within the confined transvaginal operative field. These technical demands necessitate additional training and skill refinement, contributing to the longer learning curve associated with RSP-vNOTES. These findings underscore the need for systematic training protocols and gradual case accumulation during the transition from RMP- to RSP-vNOTES procedures.

Outcomes for procedural proficiency differed between regression curve analysis and CUSUM analysis, with regression curve analysis typically requiring fewer cases to achieve proficiency. Because CUSUM applies stricter control criteria over a larger sequence of cases, it likely provides a more accurate estimate of the number of cases required to achieve proficiency. CUSUM learning curves, particularly with extended case series, offer greater precision in identifying inflection points [27]. The consistency between the two methods in our study enhances the reliability of the learning curve assessment for RSP-vNOTES hysterectomy.

For newly emerging surgical approaches, such as RSP-vNOTES, conversion and complication rates are key indicators of procedural feasibility and safety. Although operative time was used to define technical proficiency, concurrent monitoring of clinical outcomes provided essential context for evaluating safety during the learning process. A large case series of 298 RA-vNOTES procedures via the da Vinci Xi system reported a conversion rate of 1.01%. There were three conversions to robot-assisted transabdominal surgery due to unplanned bowel resection resulting from stage IV endometriosis, rectal injury, and ureteral injury [7]. The conversion rate in the present study was 1.0%, involving only one patient. The patient was converted to a transumbilical robot-assisted single-incision approach to facilitate management of diaphragmatic endometriosis identified intraoperatively; notably, the hysterectomy had been completed using the robot-assisted vNOTES approach prior to conversion. This also underscores the importance of preoperative surgical planning in identifying appropriate candidates for RSP-vNOTES. In our practice, careful patient selection is a key determinant of procedural safety when performing RSP-vNOTES hysterectomy. Specifically, candidates are selected when there is no anticipated need for bowel resection and reanastomosis requiring colorectal surgical involvement. Framing this consideration as a safety criterion, rather than a limitation of the technique itself, helps ensure appropriate application of RSP-vNOTES in clinical practice. Cases with suspected diaphragmatic disease, such as those presenting with catamenial shoulder or chest pain, hemoptysis, or a history of pneumothorax or hemothorax, as well as cases involving an extremely large uterus, are managed with traditional transumbilical robotic excision of endometriosis rather than the vNOTES approach. The overall complication rate was 14.4% (14 of 97 cases), which appears comparable to previously reported RA-vNOTES studies, where complication rates ranged from 10.0% to 16.78% [7,28].

This study does have several limitations. First, the generalizability of these findings is limited because a single, highly experienced surgeon performed all procedures, and the cohort comprises a surgically complex population. A high proportion of patients had endometriosis, many with advanced (stage III-IV) disease, which may limit direct applicability to patients undergoing hysterectomy for less complex benign conditions. Nevertheless, this case mix also demonstrates the feasibility and safety of RSP-vNOTES in technically challenging scenarios, particularly when performed by surgeons with substantial experience. Second, this is a retrospective study with inherent bias. Moreover, the observed proficiency threshold of approximately 30 cases should be interpreted in the context of the surgeon’s extensive prior experience with robotic surgery and vNOTES procedures, including more than 300 robot-assisted multi-port vNOTES cases performed using the da Vinci Xi system. As such, this threshold likely reflects adoption in experienced hands and may not be directly generalizable to surgeons earlier in their robotic learning trajectory or to those who are novice to robot-assisted vNOTES, who may require a longer learning curve to achieve similar proficiency. Lastly, the absence of a comparator or control group limits causal inference regarding postoperative outcomes, including pain improvement. Although postoperative pain scores were compared with preoperative baseline levels and the observed improvement may suggest an intervention-related effect, these findings should be interpreted descriptively rather than as definitive evidence of treatment efficacy. Additionally, the present study was designed as an observational, exploratory analysis to characterize the learning curve and procedural feasibility of RSP-vNOTES hysterectomy, rather than as a comparative effectiveness study. While comparative outcomes between RSP-vNOTES, robotic multi-port vNOTES, and conventional vNOTES have been reported in our prior work, such comparisons were beyond the scope of the current analysis [14,29].

## Conclusions

Our findings further support the safety and feasibility of RSP-vNOTES hysterectomy in expert hands. Proficiency appears achievable after 30 cases, though broader adoption may require tailored training pathways, multiple surgeons, and prospective validation.

## References

[1] Gorina Y, Elgaddal N, Weeks JD (2024). Hysterectomy among women age 18 and older. NCHS Data Brief.

[2] Harvey SV, Pfeiffer RM, Landy R, Wentzensen N, Clarke MA (2022). Trends and predictors of hysterectomy prevalence among women in the United States. Am J Obstet Gynecol.

[3] Gul G, Manzoor R, Lone IR (2024). Abnormal uterine bleeding and hysterectomy: insights from histopathological analysis of hysterectomy specimens from a tertiary care hospital. Indian J Pathol Oncol.

[4] Pillarisetty LS, Mahdy H (2023). Vaginal hysterectomy. StatPearls [Internet].

[5] (2017). Committee opinion no 701: choosing the route of hysterectomy for benign disease. Obstet Gynecol.

[6] Liu J, Tan L, Thigpen B (2022). Evaluation of the learning curve and safety outcomes in robotic assisted vaginal natural orifice transluminal endoscopic hysterectomy: a case series of 84 patients. Int J Med Robot.

[7] Yang Q, Lovell DY, Ma Y, Zhang C, Guan X (2025). The feasibility and safety of robot-assisted vaginal natural orifice transluminal endoscopic surgery (RA-vNotes) for gynecologic disease: 298-case series. Healthcare (Basel).

[8] Guan X, Lovell D, Sendukas E (2024). Pioneering case: robotic single port (SP) transvaginal NOTES (RSP-vNOTES) for hysterectomy in ten steps. Intell Surg.

[9] Zhang C, Li Q, Fang F, Wei S, Lu Q, Guan X (2024). Transvaginal NOTES hysterectomy with the Chinese robotic single port platform - report of two cases. Intell Surg.

[10] Guan X, Yang Q, Lovell DY (2024). Assessing feasibility and outcomes of robotic single port transvaginal notes (RSP-vNotes) hysterectomy: a case series. J Minim Invasive Gynecol.

[11] Santillan-Gomez A (2024). Single-port robotic-assisted transvaginal hysterectomy (vNOTES) in a hostile abdomen. Int J Gynecol Cancer.

[12] Liu Y, Fang F, Li Y (2024). Domestic robotic assisted SP vNOTES (RSP-vNOTES) for the treatment of benign gynecologic disease. Chin J Laparo Surg.

[13] Kanno K, Higuchi N, Taniguchi R, Andou M (2025). Vaginal-assisted natural orifice translumenal endoscopic surgery hysterectomy for large uterus using the da Vinci SP. J Minim Invasive Gynecol.

[14] Yang Q, Kohn J, Guan X (2025). Robotic single port versus robotic multiple port transvaginal orifice transluminal endoscopic surgery hysterectomy: a comparison of surgical outcomes. J Robot Surg.

[15] Thigpen B, Sun J, Guan X (2022). Robotic transvaginal NOTES: a step-by-step approach to surgical technique. Intell Surg.

[16] Lovell DY, Sendukas E, Yang Q, Guan X (2025). Surgical enhancement with the placement of temporary bilateral ureteral stents with indocyanine green injection for all stages of endometriosis in vNotes: retrospective cross-sectional study. J Minim Invasive Gynecol.

[17] Guan X, Yang Q, Lovell DY, Zhang C (2025). Application of "4-P" port anchoring in transvaginal natural orifice transluminal endoscopic surgery (vNOTES): technique and initial feasibility. J Robot Surg.

[18] Xu P, Zhao Z, Tian Y, Li Y, Liu Y, Ji M (2023). A retrospective analysis of robot-assisted total hysterectomy by transvaginal natural orifice transluminal endoscopic surgery. Heliyon.

[19] Mei Y, He L, Zhang Q, Chen Y, Zheng J, Xiao X, Lin Y (2023). The comparison of gasless and traditional robot-assisted transvaginal natural orifice transluminal endoscopic surgery in hysterectomy. Front Med (Lausanne).

[20] Pernar LI, Robertson FC, Tavakkoli A, Sheu EG, Brooks DC, Smink DS (2017). An appraisal of the learning curve in robotic general surgery. Surg Endosc.

[21] Maruthappu M, Duclos A, Lipsitz SR, Orgill D, Carty MJ (2015). Surgical learning curves and operative efficiency: a cross-specialty observational study. BMJ Open.

[22] Sendag F, Zeybek B, Akdemir A, Ozgurel B, Oztekin K (2014). Analysis of the learning curve for robotic hysterectomy for benign gynaecological disease. Int J Med Robot.

[23] Lin JF, Frey M, Huang JQ (2014). Learning curve analysis of the first 100 robotic-assisted laparoscopic hysterectomies performed by a single surgeon. Int J Gynaecol Obstet.

[24] Sandadi S, Gadzinski JA, Lee S (2014). Fellowship learning curve associated with completing a robotic assisted total laparoscopic hysterectomy. Gynecol Oncol.

[25] Lenihan JP Jr, Kovanda C, Seshadri-Kreaden U (2008). What is the learning curve for robotic assisted gynecologic surgery?. J Minim Invasive Gynecol.

[26] Favre A, Huberlant S, Carbonnel M, Goetgheluck J, Revaux A, Ayoubi JM (2016). Pedagogic approach in the surgical learning: the first period of “assistant surgeon” may improve the learning curve for laparoscopic robotic-assisted hysterectomy. Front Surg.

[27] Kim JK, Batra NV, Szymanski KM (2025). Evaluating the trainee impact on robotic surgery learning curves: a CUSUM analysis of large series pediatric robot-assisted laparoscopic pyeloplasty. J Robot Surg.

[28] Lowenstein L, Mor O, Matanes E, Lauterbach R, Boulus S, Weiner Z, Baekelandt J (2021). Robotic vaginal natural orifice transluminal endoscopic hysterectomy for benign indications. J Minim Invasive Gynecol.

[29] Yang Q, Lovell DY, Wu J, Zhang C, Guan X (2025). A comparative analysis of hysterectomy outcomes: robotic single-port vs. traditional transvaginal NOTES approaches. Front Med (Lausanne).

